# Facial expression, size, and clutter: Inferences from movie structure to emotion judgments and back

**DOI:** 10.3758/s13414-015-1003-5

**Published:** 2016-01-04

**Authors:** James E. Cutting, Kacie L. Armstrong

**Affiliations:** Department of Psychology, Cornell University, Uris Hall, Ithaca, NY 14853-7601 USA

**Keywords:** Clutter, Crowding, Distance Perception, Emotion, Facial Expression, Movies

## Abstract

The perception of facial expressions and objects at a distance are entrenched psychological research venues, but their intersection is not. We were motivated to study them together because of their joint importance in the physical composition of popular movies—shots that show a larger image of a face typically have shorter durations than those in which the face is smaller. For static images, we explore the time it takes viewers to categorize the valence of different facial expressions as a function of their visual size. In two studies, we find that smaller faces take longer to categorize than those that are larger, and this pattern interacts with local background clutter. More clutter creates crowding and impedes the interpretation of expressions for more distant faces but not proximal ones. Filmmakers at least tacitly know this. In two other studies, we show that contemporary movies lengthen shots that show smaller faces, and even more so with increased clutter.

The determining factor for selecting a particular shot is frequently, "Can you register the expression in the actor's eyes?" If you can't, you will tend to use the next closer shot, even though the wider shot may be more than adequate when seen on the big screen (Murch, 2001, p. 88).

Discerning other people’s expressions is likely among the more important perceptual tasks that we perform, and the literature on the perception of facial expression is vast and wide (Keltner & Ekman, 2000, for a review). We focus on the perception of valence—positive versus negative expressions, the most prominent dimension of facial emotion (Russell, 1980). Some of the neural underpinnings of this perception are clear. Adolphs, Tranel, and Damasio (1998), for example, showed that patients with bilateral lesions to their amygdalae were unable to make normal judgments of approachability from faces showing different affect. In effect, they thought that negative-valenced faces were as approachable as positive-valenced ones. Valence also affects other person-perception judgments. For example, Lander and Metcalfe (2007) found that positive-valenced faces were judged as more familiar than negative-valenced ones. This familiarity effect may contribute to the fact that positive-valenced faces also are categorized more rapidly than negative ones (Leppänen & Hietanen, 2004). Messinger, Mattson, Mahoor, and Cohn (2012) found that expressions were modulated by information in the eyes, intensifying both positive and negative emotions. After valence, intensity is the second most important dimension in facial expressions (Russell, 1980).

Our empirical focus is on how readily one can discern different emotional expressions at different distances. As straightforward as this query appears, we know of no experimental evidence that addresses it. To be sure, Euclid (Burton, 1945, p. 359) noted that “each thing has a certain limit of distance beyond which it is not longer seen.” Thus, objects at a distance become indistinct and hard to identify, and the farther a person is away the more difficult it should be to read her facial expression. The reason is that resolution will decrease with diminishing size, and spatial acuity in cycles per face (cycles per degree normalized to the size of the face) will decrease with distance (Loftus & Harley, 2004). This would suggest that in a reaction time task it might take incrementally longer to discern that expression as distance increases.

One goal of this article is to explore the relation of facial expression and distance. Other evidence aligns with this goal, and it comes from what may seem to be an unlikely quarter—popular movies. In the epigram, the academy awarding winning editor, Walter Murch, suggests why. If facial expression is important to the shot, an editor should adjust the size of the character’s face so that it is larger and can be more easily read. Our second goal is to meld the ideas of facial expression and distance with visual clutter and crowding. In particular, clutter interferes with visual search (Henderson, Chanceaux, & Smith, 2009; Williams, 1966) and may impede a task in which one must discern facial expression.

In précis, we are interested in facial scale (size coupled with distance), clutter, and the structure of movies. Studies 1 and 2 are directed at our first goal. Study 1 lays out the relationship between shot scale and shot duration for many thousands of shots in 24, and then 6, movies. It shows that shot scale and shot duration are correlated, likely because larger faces are easier and faster to read. Study 2 addresses viewers’ categorization of static facial expressions differing in scale but without clutter. Studies 3 and 4 address the second goal—exploring the effect of the recognition of expressive valence in cluttered settings. Study 3 uses stills from movies to address the categorization of facial emotion across scales in environments that have a diversity of clutter. It shows a relationship between recognition of facial expression and face size and an interaction with clutter. Study 4 analyzes the six more contemporary movies from Study 1 for their shot duration as a function of scale, clutter, and their interaction and finds results that parallel those of Study 3.

## General methods

We undertake two types of studies. Studies 1 and 4 are empirical studies about the structure of movies; Studies 2 and 3 are experiments with viewers making reaction time judgments about images. Thus, we go from movies to judgments of emotion and then back to movies to discover a heretofore unknown aspect of the physical structure of movies that is congruent with psychological data.

**Fig. 1:**
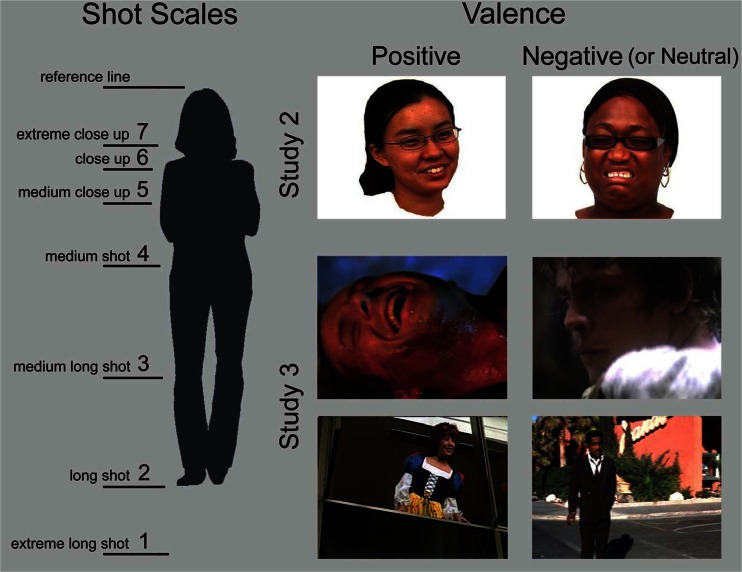
Left panel shows how shot scales are denoted and categorized. That is, what appears in the frame to the viewer at each scale lies between the reference line and the lower line that denotes each scale size. The top right panels show example stimuli used in Study 2. The bottom right panels show example stills used in Study 3 from four movies, all from DVDs. The upper ones show extreme close-ups (Scale 7) from *Die Hard 2* (Harlin, 1990, with Bruce Willis as Lt. John McClane; Twentieth Century Fox Home Entertainment), and *Star Wars: Episode V – The Empire Strikes Back* (Kershner, 1980, with Mark Hamill as Luke Skywalker; Twentieth Century Fox Home Entertainment). The lower right panels show medium long shots (Scale 3) from *Nine to Five* (Higgins, 1980, with Lily Tomlin as Violet Newstead; Twentieth Century Fox Home Entertainment) and *Ocean’s 11* (Milestone, 1960, with Sammy Davis, Jr. as Josh Howard; Warner Home Video)

The original data behind Studies 1 and 4 of movie structure come from Cutting, Brunick, and Candan (2012) but analyses here are new. We took measurements of the durations and scales all shots from 24 popular movies: 3 movies—1 drama, 1 comedy, and 1 action film—from 8 release years at 10-year intervals between 1940 and 2010, yielding a bit more than 31,000 shots. Shot duration is controlled by the number of successive frames of continuous content, begun and terminated typically by a cut, an abrupt shift of content. Shot scale is assessed by the relative size of a focal character within the frame of the movie. Cinematographically, shot scaling is a continuous measure but is typically divided into seven categories, as suggested in the left panel of Fig. 1. That is, what is between the upper reference boundary and the lower, numbered boundary for a given shot is what the viewer can see in the frame. These seven categories can be generalized to shots of other objects scaled to the size of people. Fully 90% of all shots contain characters (Cutting, 2015).

For the experiments in Studies 2 and 3, we ran 22 participants in exchange for undergraduate psychology course credit. One did not follow instructions, reversing response keys part way through the session. This left 21 viewers (19 females), all of whose data we used. Viewers participated individually in two experimental studies distributed across four computer-presented sequences in ABAB fashion. Sample stimuli are shown in the right panels of Fig. 1. Responses and stimuli from Sequences A will be called Study 2, and those from Sequences B will be called Study 3.

The analytic plan in all studies was to use least squares multiple linear regression, and occasionally stepwise regression. In both experimental studies a number of exploratory variables were considered and most of them eliminated in an effort to determine which sources of variance offered leverage into understanding the data. Reaction times (RTs) generally follow an ex-Gaussian distribution (an exponentially modified normal distribution). Therefore, all analyses (except the regression lines shown in Figs. 3 and 5 for Studies 2 and 3) were performed on inverse reaction times (1/RT) as endorsed by Ratcliff (1993). After this transformation the response distributions for our viewers were near normal, and no correct responses were discarded. All results, however, are reported as untransformed reaction times.

## Study 1: Shot scale and shot duration

### Results

**Fig. 2:**
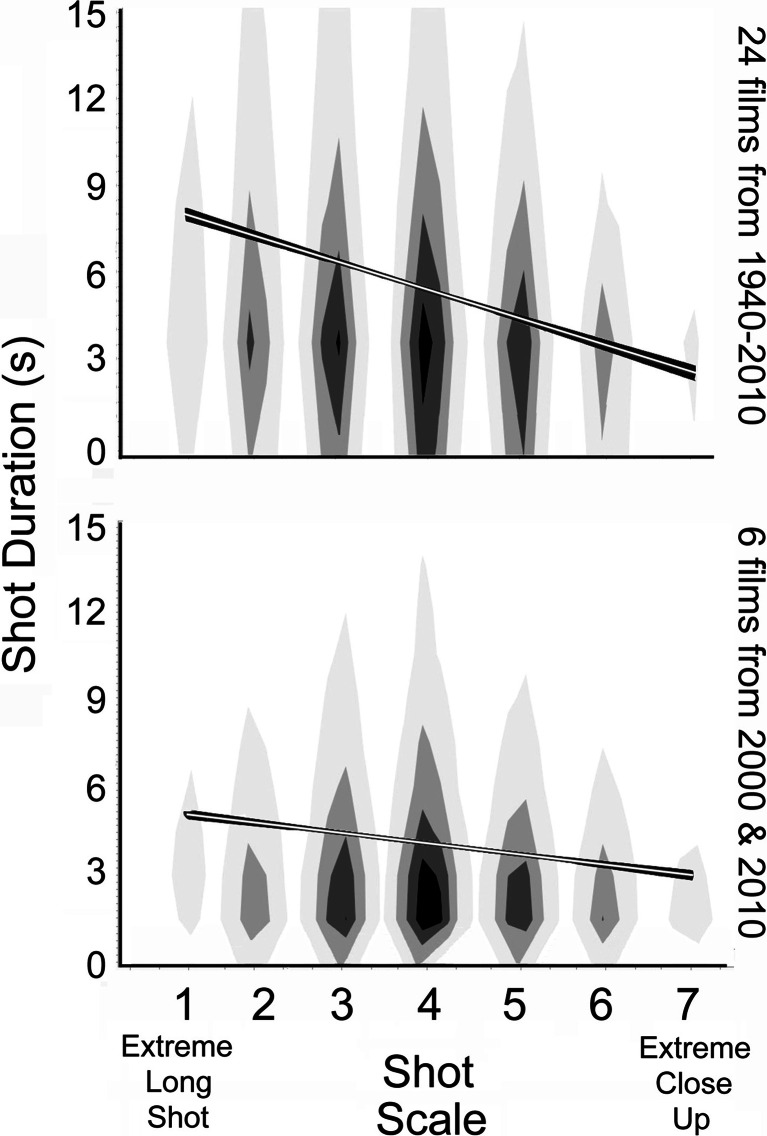
Data density clouds, regression lines, and 95% confidence intervals on the regression for the mapping of shot scale against shot duration explored in Study 1. Top panel shows the data for a bit more than 31,000 shots from 24 popular movies and the bottom panel for approximately 12,500 shots the six of those more recent movies, analyzed in Study 4. Shot scale is conventionally divided into seven categories, as shown in left panel of Fig. 1

Consider the panels of Fig. 2 where the correlation of two variables of shots in movies is assessed—shot duration versus shot scale. At each scale value, there are visual representations of the distribution of shot durations—data density clouds. Different shades of gray indicate the differing concentrations of data reflecting the whole dataset, the darkest being in regions where the density is at the 80th percentile and above for the whole distribution, the next darkest that between the 60th and 80th percentiles, and so forth, with the lowest 20% of the densities unseen and represented in white. The regression lines superimposed on these data clouds also are shown in white; darker fringes indicate the 95% confidence interval on the regression.

Notice in the top panel the sharp decline in mean shot durations as shot scale increases (*t*(31010) = −16.42, *p* < 0.0001, *d* = 0.19), with a drop from approximately 8 to 2.5 s from extreme long shots to extreme close-ups, respectively. One might suspect that this effect is due to the different narrative functions that long shots and close-ups play in movies. That is, long shots often are used as establishing shots that introduce new scenes. Because of this viewers may need more time to update their situation models concerning the narrative (Zwaan, Langston, & Graesser, 1995; Zwaan, Magliano, & Graesser, 1995; Cutting & Iricinschi, 2015), thus requiring a longer duration shot. However, when one eliminates all shots with scales below a medium close-up (Scales 1-4), and thus eliminates the likelihood of any establishing shots, there remains an undiminished correlation between shot scale and shot duration (*t*(7910) = −15.56, *p* < 0.0001, *d* = 0.35). Thus, the relation between shot scale and shot duration goes beyond any functional relationship implicated by situation models.

The data in the top panel of Fig. 2 are collapsed over seventy years of movies. The overall mean shot duration in a broader sample of 160 popular films that we have investigated has declined markedly during this period from near 14 s in the 1940s and 1950s to approximately 4.5 s in the 21st century (Cutting, Brunick, DeLong, Iricinschi, & Candan, 2011). Thus, one would expect any relation between shot duration and shot scale to become less marked, and it has. The lower panel of Fig. 2 shows data from the six films in our current sample released in 2000 and 2010. Again, a decline is prominent (*t*(12523) = −9.18, *p* < 0.0001, *d* = 0.16), although not quite so striking as before. Here, mean shot durations fall from about 4.5 s to approximately 2.5 s as scales increase from extreme long shots to extreme close ups.

### Discussion

Bordwell (2006, p. 137) reported that filmmakers understand that shorter-scaled shots (those towards close ups) can be briefer, and the data in Fig. 2 show that this relation holds. Why might this be so? Contemporary popular movies are tightly orchestrated and increasingly economical conveyors of narrative intent. Generalizing from Strunk and White (1920), every scene and every shot must tell, and tell quickly. For us, then, the most obvious reason that shot scale and shot duration might be linked is that, because movies are essentially about the emotional responses of characters to varied situations (Murch, 2001; Plantinga, 2009; Tan, 1996) and because faces are larger in shorter-scaled shots, their emotional expressions might be easier (and faster) to discern. We sought next corroborating evidence for this idea in static images, focusing on emotional valence, positive and negative. Valence, along with arousal, is considered to be a primary dimension in the conceptual space of emotions (Russell, 1980, see also Adolphs, 2002).

## Study 2: Judging emotions from faces of different sizes without clutter

### Methods

#### Stimuli

We selected 24 stimuli from a Creative Commons face sample collected, digitally manipulated, and posted by Righi, Peissig, and Tarr (2012). These are images of average young adults who, as they watched movie clips, had their expressions captured on video. Righi et al. then selected characteristic frames and removed backgrounds leaving the surrounding space white. Half of the images that we chose from this database depicted males and half females; eight were Caucasian, eight Asian-American, and eight African-American. Sample faces are shown in the top right panels of Fig. 1, and Appendix A lists the images we used as coded on the website.

Half of the selected emotional expressions denoted happiness and half disgust. In the original images, all individuals faced to the right between three-quarters profile and nearly full face. Images of half of the males and half of the females, and half expressing positive emotion and half negative emotion, were flipped around a vertical axis so that they faced to the left.

Seven stimulus variables were coded for analysis: the race of the individual (Caucasian, Asian-American, or African-American), gender, mean facial luminance, direction faced (left or right), valence of the expression (negative or positive), and whether teeth were showing. This latter variable was included because the presence of visible teeth, like information in the eyes (Messinger et al., 2012), can be interpreted as an arousal amplifier of both positive and negative affect (Calvo & Nummenmaa, 2008).

For the experimental variable of focal interest, we reproduced the faces in four sizes. For descriptive purposes, we will start by calling the largest *full size*, approximately 12° measured vertically on a computer monitor viewed at approximately 50 cm (approximately 20° is the average screen height from the center of a movie theater). The four sizes employed were full size, half size, one-quarter size, and one-eighth size. We centered the faces and formatted the stimulus frames in a 1.37 aspect ratio (width divided by height) as suggested in the top right panels of Fig. 1. In cinematographic terms, the resulting images within their frames correspond to head sizes in extreme close ups, medium shots, long shots, and extreme long shots (Scales 7, 4, 2, and 1, respectively; Fig. 1, left panel). The shot scales will be used as the independent variable of interest for statistical purposes. The 24 different faces by four sizes yielded 96 unique stimuli. Each face appeared in the center of the stimulus frame.

#### Stimulus sequence

We wrote a MATLAB Psychtoolbox-3 (Kleiner, Brainard, & Pelli, 2007) script to present the stimuli in two sequences of 192 trials each, individually randomized each time for each participant. This meant that each of the 96 stimuli were presented four times. Each sequence was preceded by instructions to respond as fast as possible with good accuracy. Viewers pressed the “f” key with their left forefinger on a QWERTY computer keyboard for negative expressions and the “j” key with their right forefinger for positive expressions. Before beginning the first test, they were given ten practice trials. Each 192-trial sequence took approximately 5 minutes to complete. All stimulus frames were presented at midscreen with no prior fixation cross. After viewers initiated the first trial, each new trial began immediately after their response. The MATLAB script allowed brief rests every 48 trials. Reaction times were collected from each viewer and all correct responses averaged.

### Results

**Fig. 3:**
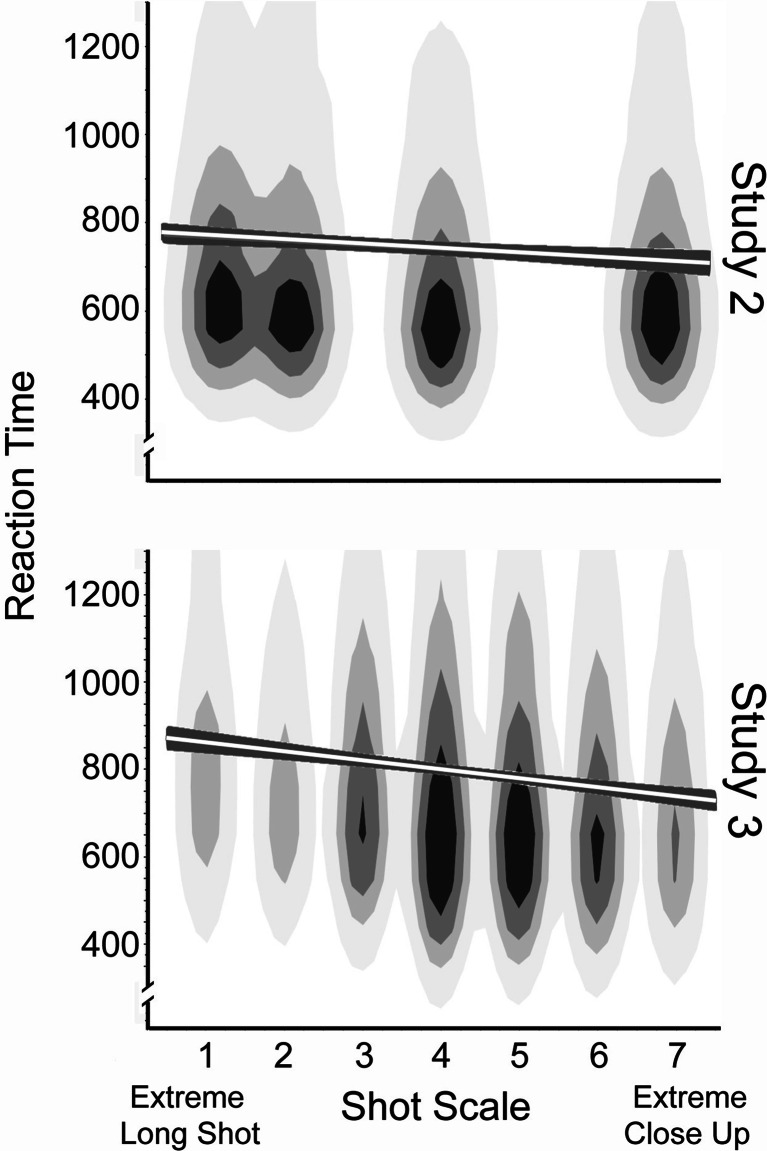
Data density clouds, regression lines, and 95% confidence intervals on the regression for reaction times in ms at four different scales in Study 2 (top panel) and at seven different scales in Study 3 (bottom panel). The interpretation of the shot scale values is given in the left panel of Fig. 1

Collapsing across viewers, scale was a competent predictor of reaction time (*t*(94) = 3.8, *p* = 0.0003, *d* = 0.78; Fig. 3, top panel). Mean reaction times were 694, 671, 645, and 641 ms, respectively, for extreme long shots (1), long shots (2), medium shots (4), and extreme close-ups (7). Notice also that the monotonic decline in response time across scales was about 50 ms. There was no reliable correlation between reaction times and error rates across all stimuli (*r* = 0.04), but errors did vary with stimulus scale (*t*(94) = 2.05, *p* = 0.021, *d* = 0.42), with error rates of 8.0%, 6.6%, 5.7%, and 6.0% across Scales 1, 2, 4, and 7, respectively.

Race, gender, facial luminance, teeth, and direction faced garnered no additional leverage in understanding the variance in the data (mean βs = 0.046). Perhaps somewhat surprisingly, there also was no effect of the valence of the emotion, although the direction of the effect (655 ms for positively valenced faces vs. 668 ms for negative) was in the direction found in the literature (Leppänen & Hietanen, 2004). We will return to valence effects in the discussion of Study 3.

Given that our research was primarily motivated by relationships among shot scales and shot durations in films, we felt that this study gave us license to seek a replication using stills taken from popular movies where we could simultaneously investigate shot scale and more naturally occurring clutter of the background.

## Study 3: Judging emotions from movie stills with more and less clutter

### Methods

#### Stimuli

Individual frames (stills) were selected from the 24 films discussed with respect to Fig. 2. See Cutting et al. (2012) or Cutting and Iricinschi (2015) for lists of those movies. A total of 330 stills were selected, from 9 to 19 per movie. Most had only one character in the frame, and if there were more only one faced the camera. Of these, 165 represented characters’ expressions in the context of the movie that corresponded to positive emotional responses and 165 that corresponded to neutral or negative emotional responses. Neutral expressions were included in the latter category on pragmatic grounds, because there simply aren’t very many negative facial expressions in popular movies. Thus, stimuli varied much more widely and evenly here in the space of expressions than did those employed Study 2.

**Fig. 4:**
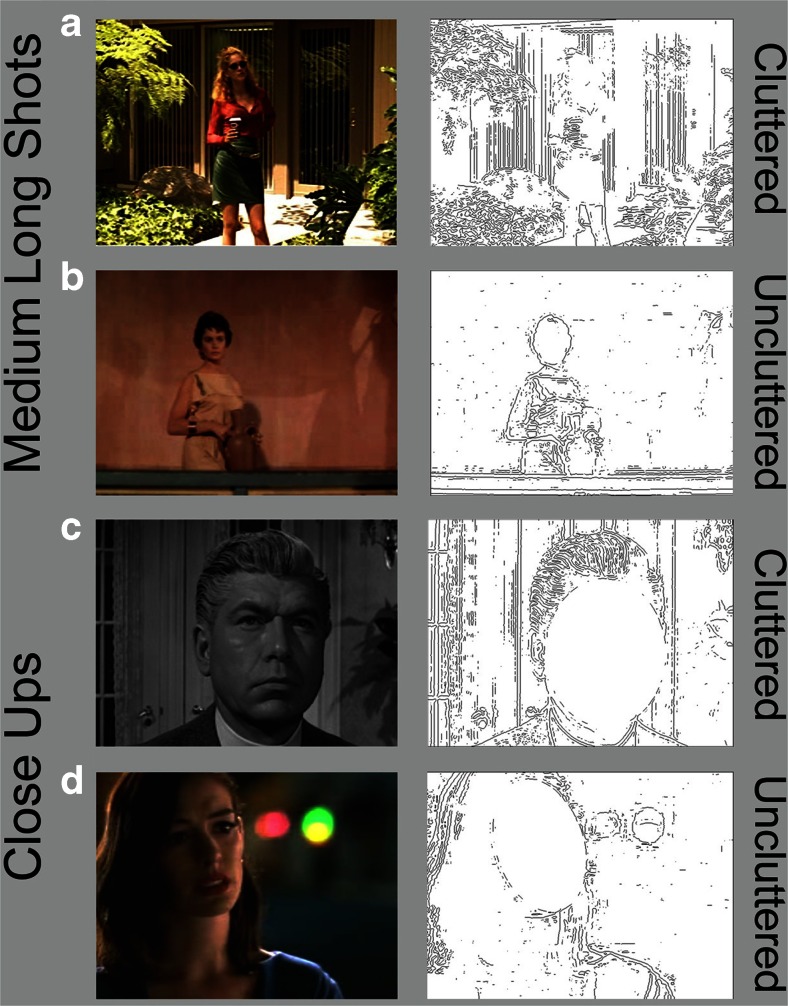
Left columns show example movie stills and the right column the luminance inverted Laplacian-of-Gaussian filtered results (with faces masked) used to compute proportional clutter. (**a, b**) Medium long shots (Scale 3). (**c, d**) Close-ups (Scale 6), all stills from DVDs. (**a**) From *Erin Brockovich* (Soderbergh, 2000, with Julia Roberts as Erin; Sony Pictures Home Entertainment). Its proportional clutter value = 0.079. (**b**) From *Spartacus* (Kubrick, 1960, with Jean Simmons as Varinia; Universal Studies Home Entertainment), clutter = 0.027. (**c**) From *Inherit the Wind* (Kramer, 1960, with Claude Akins as the Reverend Jeremiah Brown; MGM Home Entertainment), clutter = 0.067. (**d**) From *Valentine’s Day* (Marshall, 2010, with Anne Hathaway as Liz; Warner Brothers Home Video), clutter = 0.033

Darker stills were brightened and contrast enhanced so that facial expressions would be more visible. All stills appeared in an aspect ratio of 1.37, either in their original frame or trimmed from movies that were originally in formats of 1.66, 1.85, 2.2, or 2.35. Six of the films were in black-and-white, 18 in color, and stills were kept in their original achromatic or chromatic form. The proportion of stills at the different scales (0.07, 0.06, 0.15, 0.25, 0.24, 0.15, and 0.09, respectively for Scales 1 to 7) matched reasonably well their proportions among the shots in the movies from which they were taken (0.03, 0.12, 0.21, 0.35, 0.16, 0.05, and 0.01), but with some emphasis on the more extreme scales. Example stimuli are shown in the four lower right panels of Fig. 1 and the four left panels of Fig. 4.

Beyond shot scale, 12 additional stimulus variables were coded for analysis. Among these were release year of the movie from which the still was taken, whether it was in black-and-white or in color, whether it came from a shot taken outside or inside a building (to assess one aspect of lighting), and eight variables concerning the depicted character—gender, race (Caucasian vs. non-Caucasian), approximate age, valence of the expression, presence of teeth, facial angle towards or away from the camera, tilt angle of the head, and the measured distance of the center of the face from the center of the screen. A final variable concerned background clutter, a variable long known to affect visual perception and search (Ho, Scialfa, Caird, & Graw, 2001; Mack & Rock, 1998; Williams, 1966).

#### Clutter

The facial stimuli of Study 2 were devoid of backgrounds, but movie images typically have much more in them than just people and their faces. Collectively, this is called clutter, although it does not distinguish among artifacts, animals, people, textures, shadows, or anything else. In a Gestalt sense, clutter is structured ground against which a figure appears. Importantly, the amount of clutter around the character might readily attract attentional resources (Itti & Koch, 2001), making classification of the expressions more difficult. van den Berg, Cornelissen, and Roerdink (2009) distinguish two types of clutter, which we will call local density around a given object (creating a phenomenon called crowding) and global density measured throughout the frame. We will pursue this difference in discussing the results. Clutter also will vary with the lens aperture of the shot: the narrower the focal depth, the more the background will be blurred, and the less clutter will appear in the image. Thus, we felt the need to measure clutter to assess its potential effect on our results.

Clutter can be measured in a number of ways. Rosenholtz, Li, and Nakano (2007; see also Henderson et al., 2009) investigated three algorithmic approaches: feature congestion (based on the statistical saliency of image features), sub-band entropy (related to the JPEG coding of images), and edge density (based on the relative number of edges in the image). Despite showing the efficacy of all three methods predicting results in search tasks, these sets of authors did not correlate them with one another. To investigate their correlation, we digitally extracted data from their figures (Figure 9 of Rosenholtz et al., 2007; and Fig. 2 of Henderson et al., 2009) and correlated the results across the three methods. Average correlations were high (*r*s = 0.78 and 0.75, respectively, for the two articles) with edge density most highly correlated with the other two (mean *r*s = 0.85 and 0.79). Thus, we chose edge density as our measure of clutter.

We measured the edge density in all stimuli with a MATLAB script using a Laplacian of Gaussian (LoG) edge-detecting algorithm, which defines edges as zero-crossings in the image (Marr, 1982, pp. 54-78). The output is typically a black image containing a series of jagged white lines of single-pixel width. We inverted these to black-on-white, and examples are shown in right panels of Fig. 4. The black pixels correspond to locations where there is a reasonably sharp change in luminance across a region in the image. To assess the proportional clutter in the images we masked out the face of the character with a best-fitting ellipse, as shown for the unfiltered stimuli in the right panels of Fig. 6, and then measured the ratio of black to white pixels in the residual image area. Mean proportional clutter across the 330 images was 0.062 (range = 0.014 to 0.127, standard deviation = 0.018).

We also went back to the original films to fetch the durations of the shots from which these stills were taken, and analyzed the relations among shot duration, shot scale, and clutter. Shot duration was predicted by shot scale (*t*(326) = 3.53, *p* = 0.0013, *d* = 0.39), but clutter was not a factor.

#### Stimulus sequence

Each sequence had 330 stimuli and was individually randomized each time for each participant. Thus, every subject saw each stimulus twice. Each sequence took approximately 8 minutes to complete. Again, each trial began immediately after the response to the previous trial. The script allowed brief rests every 55 trials. Two reaction times were collected from each viewer for each of the 330 unique stimuli and correct responses averaged.

Again, the viewer responded to the sequences of trials driven by a purpose-written MATLAB script. The task was slightly modified in that viewers pressed the “f” key with their left forefinger on the keyboard for *neutral or negative* expressions and the “j” key with their right forefinger for positive expressions. Again, the first category was altered on pragmatic grounds, because there are very few overtly negative expressions in popular movies. Otherwise procedures and viewing conditions were the same as in Study 2.

### Results

Among the 14 independent stimulus variables in the multiple regression, 10 accounted for essentially no variance (mean βs = 0.02). Surprisingly, clutter was among these, and we will return to it below. The only reliable noncritical variable was the presence or absence of teeth in the emotional expression, where expressions showing teeth were judged more rapidly than those not (720 ms vs. 763 ms, respectively; *t*(318) = 5.31, *p* < 0.0001, *d* = 0.58). This result corroborates results of Calvo and Nummenmaa (2008); the presence of teeth is an amplifier of expression, regardless of valence, that can speed responses.

Collapsing across viewers and discarding the nonpredictive variables, we found three factors with statistical efficacy in their prediction of reaction time: scale (*t*(325) = 3.86, β = 0.20, *p* < 0.0001, *d* = 0.43), as shown in the bottom panel of Fig. 3; the measured eccentricity of the center of the face from the center of the screen (*t* = 2.4, β = 0.12, *p* = 0.017, *d* = 0.27); and the valence of the expression (*t* = 6.89, β = 0.34 *p* < 0.0001, *d* = 0.76).

Consider scale first. Mean reaction times for Scales 1 through 7 were 857, 795, 780, 716, 720, 711, and 732 ms, respectively. Notice that the effect occurs across the first six scales where the nearly monotonic decline in response time is almost 150 ms, approximately three times that of Study 2. Errors occurred on 13.4% of all trials, a value that would seem high and about which we will have more to say later. Nonetheless, there was no correlation between scale and error rate. In addition, reaction times correlated with the durations of the shots from which the stimulus stills were taken (*t*(328) = 2.47, *p* = 0.014, *d* = 0.27).

It is a little foolhardy to speculate on the cause of the upturn at the Scale 7 data. The only statistically reliable pairwise difference in the results is between the data for Scales 2 and 3. Nonetheless, Smith (2013) noticed such a turnaround in across-scales viewer correspondence of eye fixation data. That is, there was the greatest similarity and tightest correlation in eye fixations across viewers for characters portrayed in Scale 5, where observers to could see both the eyes and the mouth of the character in a single fixation, and less correspondence on other scale where either viewers either had to use separate fixations on the character’s face (Scales 6 and 7) or was free to look elsewhere on the character’s body and her surround (Scales 1-4). Perhaps here for the Scale 7 stimuli some viewers needed more than one fixation to be assured of the character’s facial expression, and this cut into the possibility of faster reaction times.

It also is possible that body cues in long-scaled shots contributed to the identification of expression valence in this study. In other words, our results might have been stronger if we could be assured that arm and body gestures of the movie characters were neutral, which almost surely was not the case. Aviezer, Trope, and Todorov (2012) found that intense positive and negative expressions were triggered more by body cues than by facial expression. Nonetheless, because we did find a reliable trend despite this possibility, we will not consider it further.

Consider next the eccentricity of the character within the frame. It makes sense that increasing eccentricity of the face from the screen center would slow judgments. In a number of cases viewers would have to execute a saccade to discern the expression. The mean eccentricity of the center of the face from the center of the screen was approximately 3° (126 pixels where the unrescaled image size was 720 by 540 pixels). Dividing the distribution at the mean eccentricity yielded mean reaction times of 719 and 766 ms for the closer and farther facial expressions, respectively.

Eccentricity also covaries with the measured size of the face in the stills. That is, smaller faces can fit farther from the center of the frame than can larger faces. We measured face size as the square root of the number of pixels in a best fitting ellipse covering the face, and this measure correlated with eccentricity across our stimulus set (*r* = 0.126, *t*(328) = 2.3, *p* = 0.022, *d* = 0.25). Thus, eccentricity needs to be considered as a covariate in other analyses. Going back to the analysis of scale, we performed stepwise regression on reaction times with eccentricity entered before scale. Results showed that even in this case scale is a more potent factor in explaining variance (*t* = 3.69, *p* = 0.0003, additional *R*^2^ = 0.039, *d* = 0.41) than is eccentricity (*t* = 2.54, *p* = 0.012, initial *R*^2^ = 0.034, *d* = 0.28).

Finally, consider valence. Leppänen and Hietanen (2004) found a positive-affect superiority for schematic faces and natural ones from the Ekman and Friesen (1976) collection. In Study 2, we found a result in the same direction, although it was not statistically reliable. Results of Study 3 would appear to fall in line with the literature, but we emphasize caution. The task and distribution of stimuli in Study 3 were different than in Study 2 and in that of Leppänen and Hietanen (2004). In those studies viewers categorized the valence of affect from strongly positive and strongly negative expressions. In Study 3, they categorized a wide array of positive expressions as separate from a wide variety of negative *and neutral* expressions. Again, the latter were included because of the paucity of negative facial expressions in popular movies. In this manner, the more continuous array of expressions and the separation of the categories in valence space different than a midpoint surely contributed to the longer reaction times here (741 ms) than in Study 2 (662 ms, *t*(40) = 9.24, *p* < 0.0001, *d* = 2.92), and greater error rates as well (13.4 vs. 6.6%). Moreover, the response to neutral and negative expressions (775 ms) was longer than the categorization of positive expressions (707 ms) as one might predict from the pair of asymmetric categories.

#### Clutter, scale, and reaction time

Again, we were surprised that proportional clutter absorbed essentially no variance in the data. However, we also were surprised that the effect of scale was three times greater here than in Study 2.

**Fig. 5:**
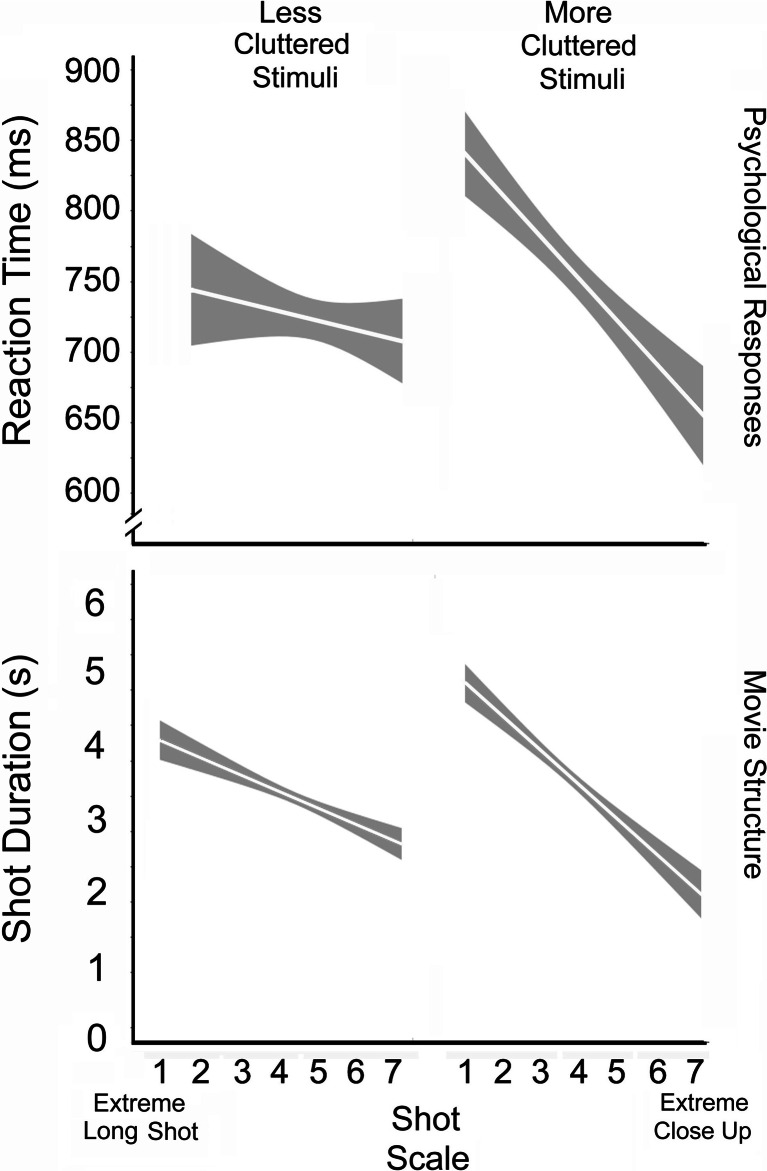
Top panel shows the interaction in Study 3 of reaction time judgments categorizing facial expressions and shot scales as a function of less (below the mean) and more (above the mean) proportional clutter. Examples of clutter are shown in right panels of Fig. 4. The bottom panel shows the interaction in Study 4 between shot duration in six movies from 2000 and 2010 and shot scales as a function of clutter (below and above the mean). How shot scales are determined is outlined in the right panel of Fig. 1. Regression lines and 95% confidence intervals are shown

Because of this large effect of scale we suspected an interaction—that is, increasing clutter in the frame might differentially slow response time. We investigated this possibility and, indeed, that is what we found. In a stepwise regression, after removing the effect of eccentricity, there was a reliable effect on reaction time of the interaction between scale and clutter (*t*(326) = 2.34, *p* = 0.01, *d* = 0.26). For display but not statistical purposes (see MacCallum, Zhang, Preacher, & Rucker, 2002) we divided the images into two groups, those above (*n* = 159) and those below (*n* = 171) the mean proportional clutter value of the stimulus set. The resulting patterns are shown in the top panel of Fig. 5.

Notice that the decline in reaction time with scale was only about 40 ms for the relatively uncluttered stills. This result is roughly comparable to what we found in Study 2 where there was no background clutter at all. On the other hand, the decline of reaction time with scale is much steeper for the more cluttered stills, fully 200 ms. The difference in these two results suggests strongly that clutter distracts viewers and renders the task more difficult (Ho et al., 2001; Williams, 1966). It also provoked our further investigation into movie structure, which is the heart of Study 4. First let us consider what clutter does for psychological processes.

#### Clutter and crowding

Clutter is the stuff more or less around the objects we are looking at or searching for. We can distinguish two kinds: global clutter distributed generally throughout the visual field, and local clutter around an object that we might be searching for. The clutter measure calculated for the stills in this study is a global measure, not distinguishing where in the frame the clutter might be. Local clutter, creating *crowding*, might be more pertinent in accounting for the interaction in the top panel of Fig. 5, making a face more difficult to find and process (see, for example, Levi, 2008). Crowding is considerably stronger in the periphery than in central vision, and since viewers typically needed to search for the face in the frame it seems a likely candidate.

**Fig. 6:**
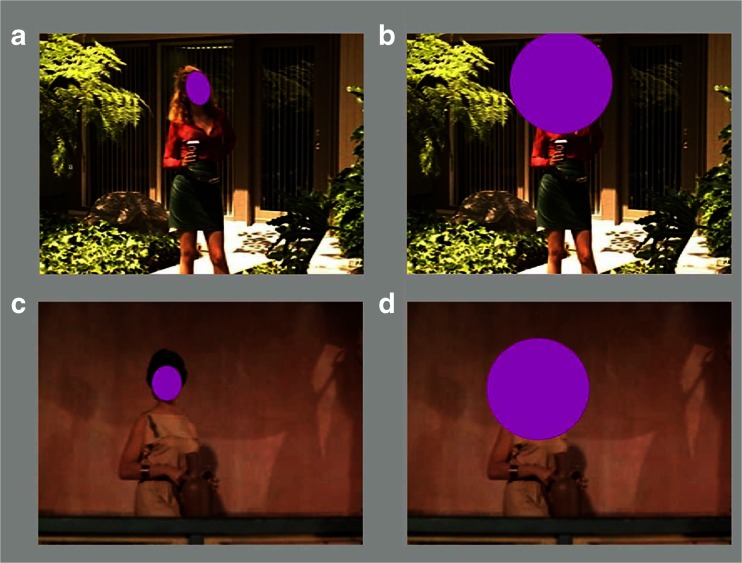
Two modifications of two stimuli from Study 3 and Fig. 4. (**a, c**) Best-fitting elliptical mask is fit over the face of the character. (**b, d**) Circular mask is centered on the face. When projected for viewers the subtense of that mask had a radius of 2.5°. LoG filter was then passed over both pairs of images and the one with the elliptical mask subtracted from that with the circular mask. The difference creates clutter index in the local area of the face, and is used to measure the effect of crowding on facial expression

To calculate crowding around the faces we first placed a best-fitting colored ellipse over the face of the character. Examples are shown in Fig. 6a and c. We then calculated the global clutter in the residual of the image using the LoG filter as before. We next created a circular mask of 2.5° radius and placed it centered over the face of the character (Fig. 6b and d). We then performed the same LoG calculation. We subtracted the latter result from the former, creating a measure of clutter that fell within the area between the face (covered by the ellipse) and the immediate surround (covered by the circle). Most Scale 5 and all Scale 6 and 7 images had faces larger than the circular mask, so they were excluded from this procedure and given a difference of zero. The array of differences became our index of crowding.

We then regressed scale, the crowding index, and the interaction of scale and crowding against reaction time. Unsurprisingly, the effect of scale returned (*t*(326) = 3.23, β = 0.23, *p* = 0.0014, *d* = 0.36). More importantly and as before, the index of crowding was not statistically reliable (β = 0.02), but the interaction between scale and crowding was (*t* = 2.45, β = 0.13, *p* = 0.015, *d* = 0.27). Thus, it is clear that local clutter and its crowding counterpart are responsible for the interaction seen at the top of Fig. 5.

#### Errors

Again, the error rate in Study 3 (13.4%) was considerably higher than in Study 2 (6.6%). As in Study 2, there was no correlation here between reactions times and error rates across stimuli (*r* = 0.011), and here none across stimuli of different scale. We suggest that many “errors” in this study are not errors at all. We followed the general tenor of the expression across the scenes of movies picking salient frames we thought would be relatively easy to categorize, but participants saw and responded only to that static image.

Each of the 330 stimuli was presented twice to each of the 21 viewers. To assess whether some stimuli were systematically miscategorized we first needed to predict the number of stimuli that would garner by chance two errors by each individual. Given that across all viewers 5,399 pairs of stimuli had no errors, and 1,191 pairs had one error, the probability of error for those 13,180 separate trials is 0.09. If errors are independent then the probability of finding two-error pairs would be total number of pairs (6,930) times the error probability twice (0.09 * 0.09) = 56.6. Given that 340 stimulus pairs had two errors, this obtained value is five times the predicted value (*z* = 11.9). Thus, it is clear that many of these stimuli did not really garner errors; instead, they were systematically categorized in opposition to our coding, with a real error rate likely closer to 9%.

## Study 4: How filmmakers accommodate scale and clutter

We know from Fig. 2 that shots with larger images of characters are generally shorter in duration than those in which their faces are smaller. What about clutter? Because many aspects of films have been changing considerably during the past 70 years (Cutting et al., 2011) and because younger viewers watch contemporary films almost exclusively, we focused on the six films (and roughly 12,500 shots) in our sample released in 2000 and 2010 and analyzed in the lower panel of Fig. 2.

### Methods

We further sampled each shot in the six films every 20 frames, yielding approximately 53,000 stills. The digital files of these movies were stored at 24 frames/s, thus samples were collected every 833 ms. Each frame was passed through a LoG filter as in Study 3 and the clutter measured as the number of pixels representing edges divided by the total number of pixels in the image. No attempt was made to mask out the characters or their faces. All results within each shot were averaged to a single value. The mean clutter measured across shots was 0.066 (standard deviation = 0.016), comparable to that for the stills in Study 3. Thus, we had for each shot in each film its duration (Cutting, DeLong, & Nothelfer, 2010; Cutting et al., 2011), its scale (Cutting et al., 2012), and an estimate of its mean clutter. We could then assess how well shot scale, shot clutter, and their interaction predicted shot duration.

## Results and discussion

Differences across shot scale predicted shot duration well, as shown for these movies in the lower panel of Fig. 2 and discussed in Study 1, but clutter did not, as in Study 3. This is likely due to the fact that clutter is strongly associated with shot scale (*t*(12514) = −36.9, *p* < 0.0001, *d* = 0.66). As noted previously, when scale value increases toward close ups the depth of focus typically decreases, blurring the background and creating less clutter. More importantly, a Shot Scale X Clutter interaction predicted shot duration quite well (*t* = −5.75, *p* < 0.0001, *d* = 0.10). Again, for display purposes only we separated all shots into those with clutter below the mean (*n* = 6,801) from those above the mean (*n* = 5,964) and in the bottom panel of Fig. 5 are plotted regression lines for mean shot scale against shot duration for the two groups. The patterns of these results are remarkably similar to those for the psychological data above it.

We know of no account in the film or media literatures that discuss anything close to this interaction among shot duration, shot scale, and clutter. Nonetheless, we conclude that, although filmmakers’ knowledge about their craft often is tacit rather than explicit, they understand that visual clutter impedes recognition, and that the longer-scale cluttered shots often must have even longer durations than they might otherwise have.

## Conclusions

Our research suggests that facial expressions are harder to read as distance increases. This is not a surprise, but the result provides empirical underpinnings for one aspect of how contemporary cinema is constructed. In particular, following Bordwell (2006), Cutting (2015) found that as shots increase the size of a character within the frame, they become shorter in duration (Fig. 2). Our results suggest that one of the reasons for the coupling of shot duration to shot scale is that facial expressions are more rapidly discerned the larger they appear on the screen. But a second coupling occurs with clutter. Although discerning expressions from larger faces seems unimpeded by background clutter, those of more distant faces are slowed beyond any difficulty in locating them away from the center of the screen. This is the effect of crowding by clutter on object identification (Levi, 2008).

Of course, even in cluttered stills the roughly 200-ms difference across scales from an extreme long shot to an extreme close-up shown at the right of the top panel of Fig. 5 cannot account for the roughly 3000-ms drop in shot duration shown in the lower right. Nonetheless, the comprehension of visual content in movies is done on much more than reading instantaneous expressions on characters’ faces; these emotions unveil over time and typically accrue while the character is talking or listening. Moreover, because it takes time for the movie viewer to orient to a new shot and to discern its content (Mital, Smith, Hill, & Henderson, 2011; Smith, 2013), we believe that it is reasonable to assume that differences in understanding as a function of shot scale and of clutter pervade visual storytelling.

More generally, we suggest that the craft of popular moviemaking is based on hard-won, practice-forged, psychological principles that have evolved over a long time, fitting stories and their presentation to our cognitive and perceptual capacities. As Münsterberg (1915, p. 31) suggested a century ago: The movie would “become more than any other art the domain of the psychologist who analyzes the working of the mind.” These particular psychologists are now called filmmakers, but we as professional psychologists can learn much from studying the structure of their products.

## Appendix A: Codes for Face Stimuli on http://wiki.cnbc.cmu.edu/Face_Place

(Righi, Peissig, and Tarr (2012)

AF0301_1100_DI.jpg, AF0303_1110_HA.jpg, AF0304_1101_HA.jpg, AF0305_1100_DI.jpg,

AM0301_1101_HA.jpg, AM0303_1100_DI.jpg, AM0304_1100_DI.jpg, AM0305_1101_HA.jpg,

BF0617_1100_DI.jpg, BF0617_2202_HA.jpg, BF0622_1101_DI.jpg, BF0623_2200_HA.jpg,

BM0613_1111_DI.jpg, BM0616_2201_DI.jpg, BM0617_1100_HA.jpg, BM0618_1100_HA.jpg,

CF0001_1101_DI.jpg, CF0002_2201_HA.jpg, CF0003_1100_DI.jpg, CF0004_1100_HA.jpg,

CM0018_1110_DI.jpg, CM0019_1110_HA.jpg, CM0026_1100_DI.jpg, CM0028_1100_HA.jpg

## References

[CR1] Adolphs R (2002). Recognizing emotion from facial expressions: Psychological and neurological mechanisms. Behavioral and Cognitive Neuroscience Reviews.

[CR2] Adolphs R, Tranel D, Damasio AR (1998). The human amygdala in social judgment. Nature.

[CR3] Aviezer H, Trope Y, Todorov A (2012). Body cues, not facial expressions, discriminate between intense positive and negative emotions. Science.

[CR4] Bordwell D (2006). The way Hollywood tells it.

[CR5] Burton HE (1945). The optics of Euclid. The Journal of the Optical Society of America.

[CR6] Calvo MG, Nummenmaa L (2008). Detection of emotional faces: Salient visual features guide effective visual search. Journal of Experimental Psychology: General.

[CR7] Cutting JE (2015). The framing of characters in popular movies. Art & Perception.

[CR8] Cutting JE, Iricinschi C (2015). Re-presentations of space in Hollywood movies. Cognitive Science.

[CR9] Cutting JE, DeLong JE, Nothelfer CE (2010). Attention and the evolution of Hollywood film. Psychological Science.

[CR10] Cutting JE, Brunick KL, DeLong JE, Iricinschi C, Candan A (2011). Quicker, faster, darker: Changes in Hollywood film over 75 years. i-Perception.

[CR11] Cutting JE, Brunick KL, Candan A (2012). Perceiving event dynamics and parsing Hollywood films. Journal of Experimental Psychology: Human Perception and Performance.

[CR12] Ekman P, Friesen WV (1976). Pictures of facial affect.

[CR13] Henderson, J. M., Chanceaux, M., & Smith, T. J. (2009). The influence of clutter on real-world scene search: Evidence from search efficiency and eye movements. *Journal of Vision, 9*(1), Article 32, 1–8. doi:10.1167/9.1.32 10.1167/9.1.32 19271902

[CR14] Ho G, Scialfa CT, Caird JK, Graw T (2001). Visual search for traffic signs: Effects of clutter, luminance, and aging. Human Factors.

[CR15] Itti L, Koch C (2001). Computational modelling of visual attention. Nature Reviews Neuroscience.

[CR16] Keltner D, Ekman P, Lewis M, Haviland-Jones J (2000). Facial expression of emotion. Handbook of emotions.

[CR17] Kleiner, M., Brainard, D., & Pelli, D. (2007). What’s new in Psychtoolbox-3? *Perception,* 36 ECVP Abstract Supplement. http://psychtoolbox.org/credits/ (Accessed 30 September 2015).

[CR18] Lander K, Metcalfe S (2007). The influence of positive and negative facial expressions on face familiarity. Memory.

[CR19] Leppänen JM, Hietanen JK (2004). Positive facial expressions are recognized faster than negative facial expressions, but why?. Psychological Research.

[CR20] Levi DM (2008). Crowding – An essential bottleneck for object recognition: A mini-review. Vision Research.

[CR21] Loftus GR, Harley EM (2004). Why is it easier to identify someone close than far away?. Psychonomic Bulletin & Review.

[CR22] MacCallum RC, Zhang S, Preacher KJ, Rucker DD (2002). On the practice of dichotomization of quantitative variables. Psychological Methods.

[CR23] Mack A, Rock I (1998). Inattentional blindness.

[CR24] Marr D (1982). Vision.

[CR25] Messinger DS, Mattson WI, Mahoor MH, Cohn JF (2012). The eyes have it: Making positive expressions more positive and negative expressions more negative. Emotion.

[CR26] Mital PK, Smith TJ, Hill RL, Henderson JM (2011). Clustering of gaze during dynamic scene viewing is predicted by motion. Cognitive Computation.

[CR27] Münsterberg, H. (1915). Why we go to the movies. *Cosmopolitan*, *60*(1)*,* 22-31. Reprinted in Corrigan, T., White, P., & Mazaj, M. (Eds.) (2010). *Critical visions in film theory* (pp. 10-16). New York: Bedford/St. Martin’s Press.

[CR28] Murch W (2001). In the blink of an eye: A perspective on film editing.

[CR29] Plantinga C (2009). Moving viewers: American film and the spectator’s experience.

[CR30] Ratcliff R (1993). Methods for dealing with reaction time outliers. Psychological Bulletin.

[CR31] Righi, G., Peissig, J. J., & Tarr, M. J. (2012). Recognizing disguised faces. *Visual Cognition, 20*(2), 143-169. doi: 10.1080/13506285.2012.654624, http://wiki.cnbc.cmu.edu/Face_Place (Accessed 30 September 2015).

[CR32] Rosenholtz, R., Li, Y., & Nakano, L. (2007). Measuring visual clutter. *Journal of Vision, 7*(2), Article 17, 1–22. doi:10.1167/7.2.17 10.1167/7.2.17 18217832

[CR33] Russell JA (1980). A circumplex model of affect. Journal of Personality and Social Psychology.

[CR34] Smith TJ, Shimamura AP (2013). Watching you watch movies: Using eye tracking to inform cognitive film theory. Psychocinematics: Exploring cognition at the movies.

[CR35] Strunk W, White EB (1920). The elements of style.

[CR36] Tan ES (1996). Emotion and the structure of narrative film: Film as an emotion machine.

[CR37] van den Berg, R., Cornelissen, F. W., & Roerdink, J. B. T. M. (2009). A crowding model of visual clutter. *Journal of Vision, 9*(4), .Article 24, 1–7. doi:10.1167/9.4.24 10.1167/9.4.24 19757933

[CR38] Williams LG (1966). The effect of target specification on objects fixated during visual search. Attention, Perception, & Psychophysics.

[CR39] Zwaan RA, Langston MC, Graesser AC (1995). The construction of situation models in narrative comprehension: An event-indexing model. Psychological Science.

[CR40] Zwaan RA, Magliano JP, Graesser AC (1995). Dimensions of situation model construction in narrative comprehension. Journal of Experimental Psychology: Learning, Memory, and Cognition.

[CR41] Harlin, R., director. (1990). *Die Hard 2.* USA: Twentieth Century Fox.

[CR42] Higgins, C., director. (1980). *Nine to Five.* USA: Twentieth Century Fox.

[CR43] Kershner, I., director. (1980). *Star Wars: Episode V - The Empire Strikes Back.* USA: Lucasfilm.

[CR44] Kramer, S., director. (1960). *Inherit the Wind.* USA: United Artists.

[CR45] Kubrick, S., director. (1960). *Spartacus.* USA: Universal Pictures.

[CR46] Marshall, G., director. (2010). *Valentine’s Day.* USA: New Line Cinema.

[CR47] Milestone, L., director. (1960). *Ocean’s 11.* USA: Warner Brothers.

[CR48] Soderbergh, S., director. (2000). *Erin Brockovich.* USA: Universal Pictures.

